# Semi-automated thrombin dynamics applying the ST Genesia thrombin generation assay

**DOI:** 10.3389/fcvm.2022.912433

**Published:** 2022-07-26

**Authors:** Audrey Carlo, Qiuting Yan, Hugo Ten Cate, Romy De Laat-Kremers, Bas De Laat, Marisa Ninivaggi

**Affiliations:** ^1^Diagnostica Stago S.A.S., Asnières-sur-Seine, France; ^2^Department of Functional Coagulation, Synapse Research Institute, Maastricht, Netherlands; ^3^Department of Biochemistry, Maastricht University, Maastricht, Netherlands; ^4^Department of Data Analysis and Artificial Intelligence, Synapse Research Institute, Maastricht, Netherlands

**Keywords:** thrombin generation, thrombin dynamics, antithrombin, fibrinogen, oral contraceptives

## Abstract

**Background:**

The haemostatic balance is an equilibrium of pro- and anticoagulant factors that work synergistically to prevent bleeding and thrombosis. As thrombin is the central enzyme in the coagulation pathway, it is desirable to measure thrombin generation (TG) in order to detect possible bleeding or thrombotic phenotypes, as well as to investigate the capacity of drugs affecting the formation of thrombin. By investigating the underlying processes of TG (i.e., prothrombin conversion and inactivation), additional information is collected about the dynamics of thrombin formation.

**Objectives:**

To obtain reference values for thrombin dynamics (TD) analysis in 112 healthy donors using an automated system for TG.

**Methods:**

TG was measured on the ST Genesia, fibrinogen on the Start, anti-thrombin (AT) on the STA R Max and α_2_Macroglobulin (α_2_M) with an in-house chromogenic assay.

**Results:**

TG was measured using STG-BleedScreen, STG-ThromboScreen and STG-DrugScreen. The TG data was used as an input for TD analysis, in combination with plasma levels of AT, α_2_M and fibrinogen that were 113% (108–118%), 2.6 μM (2.2 μM−3.1 μM) and 2.9 g/L (2.6–3.2 g/L), respectively. The maximum rate of the prothrombinase complex (PCmax) and the total amount of prothrombin converted (PCtot) increased with increasing tissue factor (TF) concentration. PC_tot_ increased from 902 to 988 nM, whereas PC_max_ increased from 172 to 508 nM/min. Thrombin (T)-AT and T-α_2_M complexes also increased with increasing TF concentration (i.e., from 860 to 955 nM and from 28 to 33 nm, respectively). PC_tot_, T-AT and T-α_2_M complex formation were strongly inhibited by addition of thrombomodulin (−44%, −43%, and −48%, respectively), whereas PC_max_ was affected less (−24%). PC_tot_, PC_max_, T-AT, and T-α_2_M were higher in women using oral contraceptives (OC) compared to men/women without OC, and inhibition by thrombomodulin was also significantly less in women on OC (*p* < 0.05).

**Conclusions:**

TG measured on the ST Genesia can be used as an input for TD analysis. The data obtained can be used as reference values for future clinical studies as the balance between prothrombin conversion and thrombin inactivation has shown to be useful in several clinical settings.

## Introduction

The haemostatic balance is an equilibrium of pro- and anticoagulant factors that work synergistically to maintain haemostasis and prevent bleeding and thrombosis. When coagulation is triggered, both intrinsic and extrinsic coagulation pathways come together in one common pathway that finally leads to the generation of thrombin (1). Thrombin is the key player of the coagulation cascade as it not only cleaves fibrinogen into fibrin monomers, but also evokes the activation of coagulation factors (F) V, VIII, and XI, and the protease activated receptors that are present on platelets (2, 3).

As thrombin has various roles in the regulation of the coagulation cascade, it is desirable to measure thrombin generation (TG) in order to detect possible bleeding or thrombotic phenotypes. Thrombin generation measured with the semi-automated Calibrated Automated Thrombinography (CAT) method has been shown to be associated with the risk of bleeding and thrombosis in clinical studies (4–10). It is a sensitive tool to identify congenital or acquired haemostatic disorders, and can be used to investigate the efficacy of drugs that affect the formation of thrombin [3, 6]. Nowadays, the ST Genesia performs a fully automated TG measurement in platelet poor plasma (11–13).

Several years ago, the thrombin dynamics (TD) analysis was developed for the CAT method (14). This computational method allows the researcher to study the pro- and anticoagulant processes that determine TG: prothrombin conversion and thrombin inactivation. Over the past years, the TD method has shown its usefulness in multiple clinical settings (15–23). TD analysis can pinpoint changes in prothrombin conversion or thrombin inactivation that provoke a change in coagulation. In liver cirrhosis patients, TD analysis provided evidence that indeed TG is rebalanced and that the prothrombin conversion and thrombin inactivation are both reduced due to lower coagulation factor production (19). Moreover, as the TD method partially relies on the computational modeling of the inactivation of thrombin, *in silico* experimentation can be used to predict the effect of changes in prothrombin conversion and thrombin inactivation on the TG curve. *In silico* modeling was used in several clinical settings, for example to study the effect of prothrombin complex concentrate administration with and without added antithrombin (AT) after cardiopulmonary bypass surgery (17), to predict the effect of AT expression targeting in hemophilia patients and investigate inter-patients variability (24), and in trauma patients (25).

As TD analysis has been shown to be clinically relevant in a research setting, we set out to perform TD analysis as previously described,(14) using TG curves acquired on the newly developed ST Genesia (Diagnostica Stago, Asnières-sur-Seine, France) (26). The ST Genesia is a benchtop, fully automated TG assay, related to the previous CAT assay, which is available in clinical laboratories. The use of the ST Genesia for TG has the advantage that it is a more standardized method and has specific reagent kits for specific experimental aims. Differences between the CAT and ST Genesia method, is that the latter one includes the presence of a reference plasma and quality controls in the reagent kits and has a different calibration method (13). We determined reference values for all ST Genesia acquired TD parameters using all currently available ST Genesia reagents (STG-BleedScreen, STG-ThromboScreen and STG-DrugScreen) in 112 healthy donors. These reference ranges can be used for comparison by other research groups after verification in healthy volunteers according to the CLSI guidelines (27). Moreover, we investigated the effect of sex and oral contraceptive (OC) use on TD parameters.

## Materials and methods

### Blood collection

The population described in this study was previously investigated in a study by Ninivaggi et al. (13). In total, 112 healthy donors were included in this study. The study was conducted according to the Declaration of Helsinki (2013) and approved by the Medical Ethical Committee of the Maastricht University Medical Center. Non-inclusion criteria for this study were the use of drugs interfering with coagulation, having a known coagulation disorder and/or being younger than 18 or older than 65 years. Blood samples were taken from the donors only after signing the informed consent. Vacuum blood drawing tubes (Greiner Bio-One) containing 3.2% sodium citrate (in a 9:1 ratio blood:citrate) were used to draw the blood from the antecubital vein. To reduce the effect of pre-analytical factors on TG, the tourniquet was only used for the purpose of finding the vein, but loosened afterwards, and the first tube of blood was discarded. Platelet poor plasma was obtained by centrifuging the blood twice for 10 min at 2630 g at room temperature immediately after blood drawing. The anonymized plasma samples were stored directly at −80°C until further use.

### Thrombin generation

The ST Genesia was used to measure TG as previously described (13). In short, first a calibration curve was performed by using the STG-Cal&Fluo kit. Once the calibration has been performed successfully, the reference plasma and quality controls were measured. For this study three reagent kits were used that are commercially available: STG-BleedScreen, STG-ThromboScreen and STG-DrugScreen. Each reagent kit contains its own reference plasma that is used to normalize the sample TG data, as well as 2 or 3 quality controls. The STG-ThromboScreen reagent kit contains two activators with the same TF and phospholipid content, but one of them also contains TM purified from rabbit lung. After successful completion of these runs, the plasma samples of the healthy donors were thawed in a warm water bath at 37°C for 5 min. Immediately hereafter, the plasma tubes were gently mixed, placed on board and TG was measured. The samples were run in duplicate and the ST Genesia embedded software analyzed the data automatically and gave a mean result for the duplicates. The reference plasma run in parallel to the samples, comes with specific assigned ranges provided on a barcoded flier and helps to normalize results (26, 28). The ST Genesia normalizes the results for each sample automatically by applying the following formula: [patient sample result / reference plasma result **·** activity assigned for the particular lot and parameter of this reference plasma].

### Plasma levels of antithrombin, fibrinogen, and α_2_Macroglobulin

AT, fibrinogen and α_2_Macroglobulin (α_2_M) levels were measured to perform TD analysis. AT was measured on the automated coagulation analyzer STA-R Max according to manufacturer specifications using STA^®^-Stachrom ATIII kit (reagent and analyzer Diagnostica Stago, Asnières-sur-Seine, France). Functional fibrinogen levels were measured using the Clauss method with STA^®^-Liquid Fib on STart (reagent and semi-automated analyzer Diagnostica Stago, Asnières-sur-Seine, France). Plasma α_2_M levels were measured with an in-house chromogenic assay as previously described by Kremers et al. (14).

### Thrombin dynamics

The TG curve is the net result of prothrombin conversion and subsequent thrombin inactivation (14). The course of prothrombin conversion can be calculated if the thrombin concentration at a certain time point (i.e., a point on the TG curve) and the rate of thrombin inactivation are known. The inactivation of thrombin is a relatively simple process, in which only a few mediators have a clinically meaningful effect. During TG, thrombin is inactivated mainly by AT, and a smaller fraction is inhibited by α_2_M and a group of various minor inhibitors. Additionally, the fibrinogen content of a plasma sample has been shown to influence the rate of thrombin inhibition, because fibrin(ogen) binds and thereby protects thrombin from inhibition by its natural inhibitors (14, 29).

### Computation of thrombin inactivation

Several years ago, we developed a computational model that predicts the rate of inactivation of a certain amount of thrombin based on the plasma concentrations of AT, α_2_M and fibrinogen (14, 17, 18, 30). This model consists of a set of ordinary differential equations, which describe the rate of thrombin inactivation in time based on the plasma AT, α_2_M and fibrinogen level and the concentration of free thrombin at each point in time (Equations 1–3).


(1) $$\begin{array}{r} \text{d}\left(\text{T}-\text{AT}\right)/\text{dt}={\text{k}}_{\text{AT}}\cdot {\left[\text{AT}\right]}_{\text{t}}\cdot \;{\left[{\text{T}}_{\text{free}}\right]}_{\text{t}} \end{array}$$

(2) $$\begin{array}{r} \text{d}\left(\text{T}-{\text{α}}_{2}\text{M}\right)/\text{dt}={\text{k}}_{\text{α}2\text{M}}\cdot {\left[{\text{α}}_{2}\text{M}\right]}_{\text{t}}\cdot \;{\left[{\text{T}}_{\text{free}}\right]}_{\text{t}} \end{array}$$

(3) $$\begin{array}{l} \begin{array}{c} -\text{d}({\text{T}}_{\text{free}})/\text{dt}={\text{k}}_{\text{AT}}\cdot {\left[\text{AT}\right]}_{\text{t}}\cdot {\left[{\text{T}}_{\text{free}}\right]}_{\text{t}}+ \end{array}\\ \begin{array}{c} {\text{ k}}_{\text{α}2\text{M}}\cdot {\left[{\text{α}}_{2}\text{M}\right]}_{\text{t}}\cdot \;{\left[{\text{T}}_{\text{free}}\right]}_{\text{t}} \end{array} \end{array}$$

The amount of free thrombin (T_free_) depends on the amount of thrombin substrate that is present, and rate constants for the inactivation of thrombin by antithrombin (k_AT_) and α_2_-macroglobulin (k_αM_) are dependent on the plasma fibrinogen level, as described in more detail elsewhere (14).

### Computation of prothrombin conversion

As previously described, this computational model can be used to extract prothrombin conversion curves from TG data (23). At any moment during the course of the TG process, the thrombin concentration (i.e., the TG curve) is the net result of prothrombin conversion and thrombin inactivation. As a result, the course of prothrombin conversion (d(P)/dt) can be calculated from the TG curve ([T]t) if the inactivation rate of thrombin for a specific thrombin concentration (d(T-inh)/dt) is known (Equation 4). We can calculate the thrombin inactivation rate at each time point during TG, using the model for thrombin inactivation as described above (Equation 5), and the thrombin concentration obtained from the thrombin generation curve. Additionally, experimentally determined plasma levels of AT, α_2_M and fibrinogen are required as an input for the computational model.


(4) $$\begin{array}{r} \text{d}\left(\text{T}\right)/\text{dt}=-\text{d}\left(\text{P}\right)/\text{dt}-\text{d}\left(\text{T}-\text{inh}\right)/\text{dt} \end{array}$$

(5) $$\begin{array}{l} \begin{array}{c} -\text{d}(\text{P})/\text{dt}=\text{d}(\text{T})/\text{dt}+{\text{k}}_{\text{AT}}\cdot {\left[\text{AT}\right]}_{\text{t}}\cdot {\left[\text{T}\right]}_{\text{t}}+ \end{array}\\ \;\begin{array}{c} {\text{k}}_{\text{α}2\text{M}}\cdot {\left[{\text{α}}_{2}\text{M}\right]}_{\text{t}}\cdot \;{\left[\text{T}\right]}_{\text{t}} \end{array} \end{array}$$

Similar to the TG curve, the prothrombin conversion curve is typically quantified by several parameters: PC_tot_ (total amount of prothrombin converted during the TG test; area-under-the-curve), PC_max_ (maximum rate of prothrombin conversion, peak height of the curve), T-AT (amount of thrombin-antithrombin complexes formed), and T-α_2_M (amount of thrombin-α_2_M complexes formed) (14, 23). Additionally, the total inhibitory potential of each plasma is quantified by the thrombin decay constant (TDC), independent of prothrombin conversion, and solely based on the levels of AT, α2M, and fibrinogen (23). The thrombin decay capacity is the pseudo-first order decay constant for thrombin that combines the overall effect of thrombin inactivation by AT and α_2_M, and the interference of fibrinogen in this process.

### High throughput thrombin dynamics analysis

Thrombin dynamics analysis was performed in Matlab (R2016a, Mathworks, Eindhoven, the Netherlands) using the Matlab “Statistics and Machine Learning Tool Box” (version 10.2, Mathworks, Eindhoven), and theMatlab “Curve Fitting Toolbox” (version 3.5.3, Mathworks, Eindhoven, the Netherlands) and to ensure a high throughput computation of thrombin dynamics parameters. Analyses were performed on a HP probook (HP, Amstelveen, the Netherlands) equipped with a Microsoft Windows 10 pro operating system. The equations described in the sections above were implemented in an semi-automized data analysis script, which analyzes around 50 curves per minute.

### Statistics

The GraphPad Prism software (version 8.4.2, San Diego, CA) was used to determine statistical significance of the results. Data are presented as median ± interquartile range (IQR) as indicated. Data were checked for normality by the Shapiro-Wilk test. As not all groups passed normality, the Friedman test was used to compare paired data in combination with the Dunn's *post-hoc* test for multiple comparison. Similarly, for unpaired data, the Kruskal-Wallis test was used in combination with Dunn's post hoc test for multiple comparison. Reference ranges were established by calculating the 2.5th and 97.5th percentile in the dataset of 112 healthy subjects. A *p*-value < 0.05 was considered statistically significant.

## Results

### Thrombin generation measured on the ST Genesia

TG was measured using the three reagent kits available for the ST Genesia: STG-BleedScreen (*N* = 112), STG-ThromboScreen (*N* = 112) and STG-DrugScreen (*N* = 111). The TF concentrations of the aforementioned reagent kits are manufacturer proprietary information, however, they range from low to medium and high, respectively. Table 1 summarizes the following TG parameters: lag time, peak height, time-to-peak, Endogenous Thrombin Potential (ETP), velocity index for all reagent kits, and ETP inhibition induced by TM for STG-ThromboScreen kit. As expected, peak height and velocity index increased with increasing TF concentration, while lag time and time-to-peak shorted with increasing TF concentration. The effect on ETP was less pronounced compared to the other parameters. Addition of TM affected especially peak height and ETP, and to a minor extent velocity index, lag time and time-to-peak. The median (IQR) ETP inhibition was 47% (34.9–64.3%).

**Table 1:** Thrombin generation data.

	**STG-BleedScreen**		**STG-ThromboScreen**		**STG-DrugScreen**	
	**Median**	**IQR**	**Median**	**IQR**	**Median**	**IQR**
Lagtime (min)	2.9	2.6–3.4	2.3	2.1–2.6	1.3	1.2–1.5
Peak (nM)	168	140–204	195	169–238	347	300–405
Time-to-peak (min)	6.4	5.8–7.3	5.4	4.6–6.2	2.8	2.5–3.3
ETP (nM*min)	1112	934–1,325	1,127	993–1,388	1213	1,087–1,419
Velocity Index (nM/min)	60	45–81	84	63–115	328	231–417

*TG was measured with the ST Genesia using the STG-BleedScreen (N = 112), STG-DrugScreen (N = 111) and STG-ThromboScreen (N = 112). Data are median with IQR. ETP, endogenous thrombin potential; TM, thrombomodulin; IQR, interquartile range*.

### Thrombin dynamics on ST Genesia TG data

The TG data generated on the ST Genesia was used as an input for TD analysis, in combination with the plasma levels of AT, α_2_M and fibrinogen that were measured for each subject. The median values (IQR) for AT, α_2_M and fibrinogen were 113% (108–118%), 2.6 μM (2.2–3.1 μM), and 2.9 g/L (2.6–3.2 g/L), respectively.

**Figure 1:**
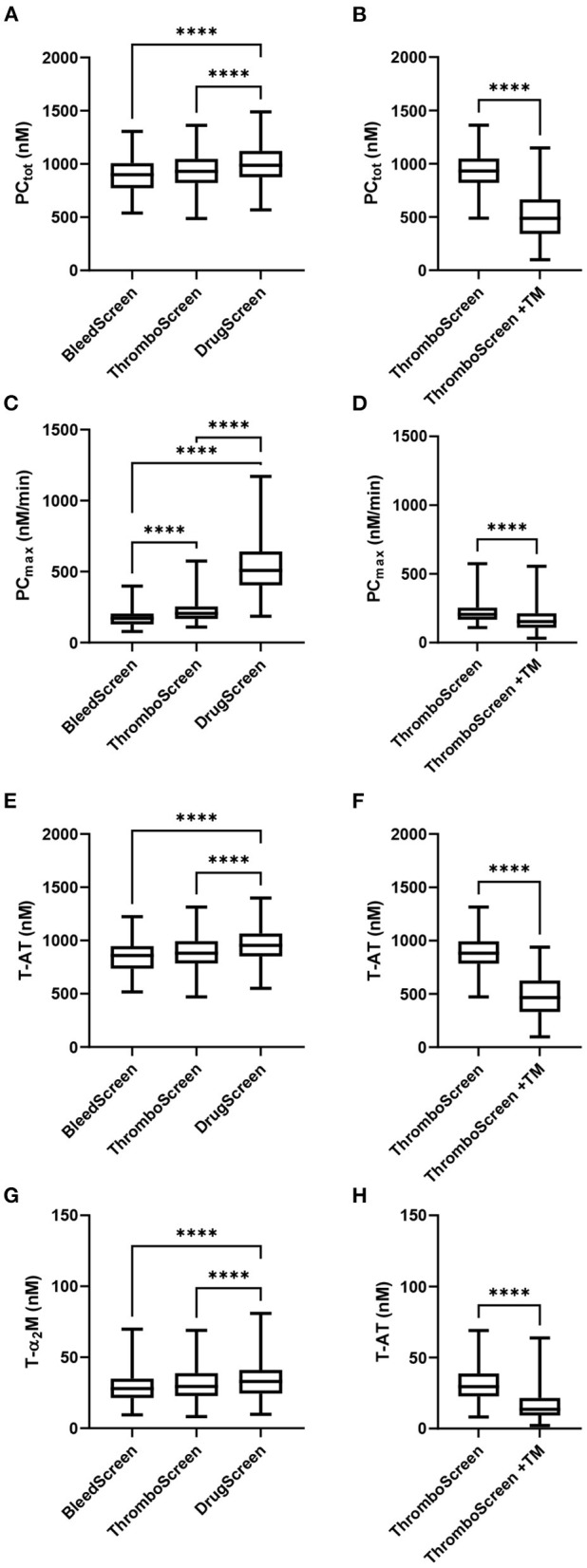
Thrombin dynamics parameters obtained from thrombin generation data measured with the ST Genesia. TD analysis was performed on the TG data measured with the STG-BleedScreen(*N* = 112), STG-DrugScreen (*N* = 111), and STG-ThromboScreen with and without TM (*N* = 112). TD parameters PCtot **(A,B)**, PCmax **(C,D)**, T-AT **(E,F)**, and T-α_2_M **(G,H)** complexes are depicted. Data are median with interquartile ranges and minimum and maximum values. tot, total; max, maximum; TM, thrombomodulin; T-AT, thrombin-anti-thrombin complex; T-α_2_M, thrombin-α_2_M complex. ****P ≤ 0.0001.

The TD parameters PC_tot_, PC_max_, T-AT, and T-α_2_M are shown in Figure 1. PC_tot_, increased slightly with increasing TF concentration from 902 nM to 933 nM to 988 nM with the STG-BleedScreen, STG-ThromboScreen and STG-DrugScreen, respectively (Figure 1A). On the contrary, PC_max_ increased strongly with increasing TF concentration and ranged from 172 to 206 nM/min to 508 nM/min for the aforementioned reagent kits (Figure 1C). Similarly to the ETP, T-AT, and T-α_2_M complexes increased with the TF concentration (Figures 1E,G). T-AT was, respectively for the STG-BleedScreen, STG-ThromboScreen and STG-DrugScreen, 860, 897, and 955 nM, while T-α_2_M was 28, 30, and 33 nM. The effect of TM was measured using the STG-ThromboScreen kit, which as previously described contains TG trigger with and without TM. PC_tot_, T-AT, and T-α_2_M complex formation are strongly inhibited by the addition of TM (−44, −43, and −48%, respectively, Figures 1B,F,H), whereas PC_max_ was affected less, but still significantly (−24%, Figure 1D).

The reference values for each TD parameter and STG reagent were calculated as the 2.5th and 97.5th percentile of the distribution using the data from all 112 healthy donors and are shown in Table 2.

**Table 2:** Reference ranges for thrombin dynamics parameters determined on the ST Genesia.

	**Median**	**2.5th percentile**	**97.5th percentile**
**STG-BleedScreen**			
PC_tot_ (nM)	902	609	1239
PC_max_ (nM/min)	172	90	364
TAT (nM)	860	586	1,182
Ta_2_M (nM)	28	12	60
**STG-DrugScreen**			
PC_tot_ (nM)	988	654	1,416
PC_max_ (nM/min)	508	232	1,088
TAT (nM)	955	630	1,364
Ta_2_M (nM)	33	17	59
**STG-ThromboScreen**			
PC_tot_ (nM)	933	542	1,251
PC_max_ (nM/min)	206	115	472
TAT (nM)	897	526	1,202
Ta_2_M (nM)	30	15	56
**STG-ThromboScreen +TM**			
PC_tot_ (nM)	492	188	1,001
PC_max_ (nM/min)	153	56	466
TAT (nM)	470	184	870
Ta_2_M (nM)	14	4	41

*TG was measured with STG-BleedScreen, STG-DrugScreen, and STG-ThromboScreen on the ST Genesia in 112 healthy subjects and reference ranges were calculated as the 2.5th and 97.5th percentile of the distribution*.

### Effect of sex and oral contraceptive use

It has been previously reported that TG and TD parameters (23) differ between men and women, and that the use of OC can influence TG in women (11, 13, 31). Here, we compared three groups: men, women without the use of OC and women taking OC. No significant differences in TG parameters between men and women without OC were found whatever the trigger reagent, except for the lag time, that was consistently shorter in women (Supplementary Table 1). On the contrary, women taking OC showed not only a shorter lag time and time-to-peak, but also a higher peak height and ETP, compared to women that did not use OC or men. A more pronounced consequence of OC usage could be observed after addition of TM as ETP inhibition in women using OC shows a median value of 25.5% only (22.3–32.3%) compared to 57.6% (43.4– 65.8%; *p* < 0.0001) and 45.0% (34.9– 63.3%; *p* = 0.0006) in men and women without OC, respectively.

**Figure 2:**
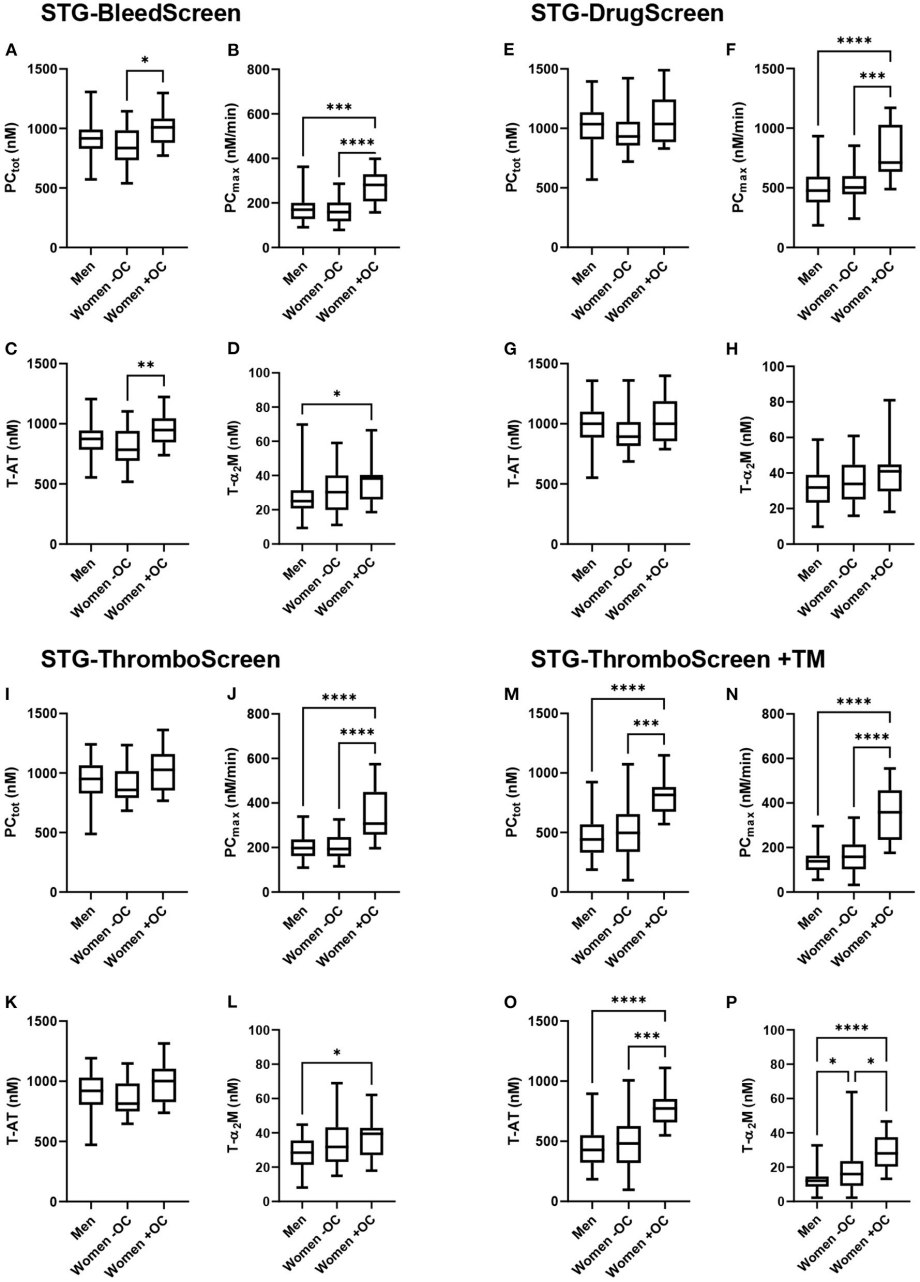
Thrombin dynamics parameters obtained from thrombin generation measured with the ST Genesia stratified for sex and oral contraceptive use. TG was measured with the STG-BleedScreen **(A–D)**. STG-DrugScreen **(E–H)**, STG-ThromboScreen without TM **(I–L)**, and STG-ThromboScreen with TM **(M–P)**. TD parameters PCtot **(A,E,I,M)**, PCmax **(B,F,J,N)**, T-AT **(C,G,K,O)**, and T-α_2_M **(D,H,L,P)** complexes are depicted for each group: men, women without OC and women with OC. Data are median with IQR and minimum and maximum values. tot, total; max, maximum; T-AT, thrombin-antithrombin complex; T-α_2_M, thrombin-α_2_M complex; TM, thrombomodulin. *P ≤ 0.05, **P ≤ 0.01, ***P ≤ 0.001, ****P ≤ 0.0001.

TD parameters were compared between men, women without OC and with OC for all ST Genesia reagents (Figure 2). PC_tot_ did not differ between men and women without OC, regardless of the reagent used. However, PC_tot_ was significantly higher in women using OC compared to women without OC if TG was measured with the STG-BleedScreen reagent (+21%; *p* = 0.017), and in the presence of TM (STG-ThromboScreen+TM reagent; +64 %; *p* < 0.001). T-AT complexes followed the same trend as PC_tot_: no difference was observed between men and women, but women on OC showed a higher T-AT formation than women without OC, for the STG-BleedScreen (948 vs. 784 nM; *p* = 0.009) and STG-ThromboScreen+TM (773 vs. 482 nM; *p* = 0.001) reagents.

PC_max_ was comparable between men and women without OC, but significantly higher in women using OC than in men, regardless of the trigger used (+49% up to +158% depending on the trigger reagent, all *p* < 0.001). Also, compared to women without OC, PC_max_ was significantly higher (+41% up to +127% depending on the trigger reagent, all *p* < 0.001). T-α_2_M formation was significantly higher in women using OC than men for the STG-BleedScreen (+52%, *p* = 0.04), STG-ThromboScreen (+39%, *p* = 0.04), and STG-ThromboScreen+TM (+133%, *p* < 0.001). Furthermore, in the presence of TM, T-α_2_M formation was significantly higher in women without OC compared to men (+33%, *p* = 0.04), and in women using OC compared to women without OC (+75%, *p* = 0.01).

**Figure 3:**
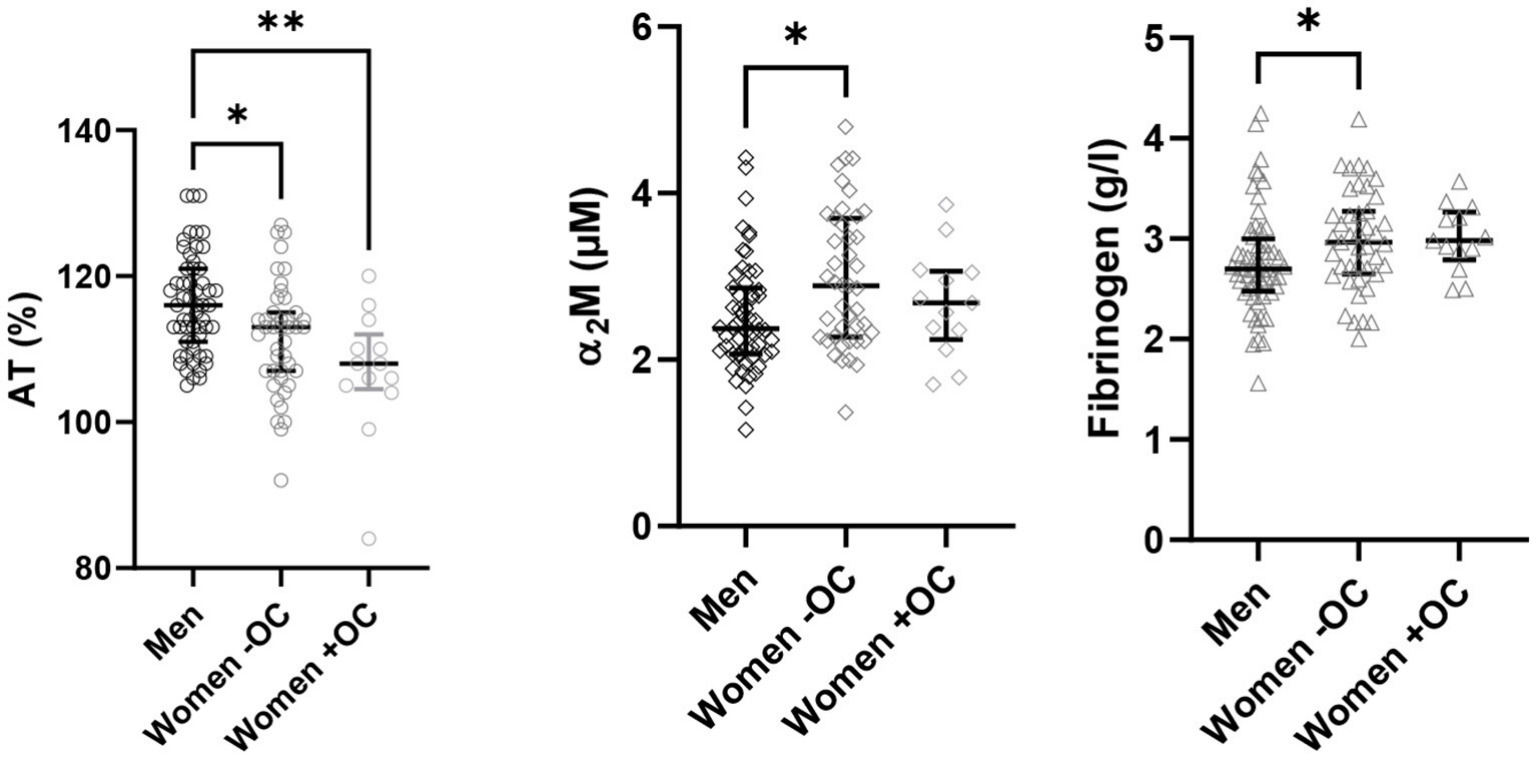
Antithrombin, α_2_M and fibrinogen levels. The coagulation factor levels were measured in the plasma of 112 healthy donors and are depicted for the three groups: men, women without OC and women with OC. Data are median with IQR. AT, antithrombin; OC, oral contraceptives. *P ≤ 0.05, **P ≤ 0.01.

We further investigated whether differences AT, α_2_M and fibrinogen could explain the differences in TD parameters in men and women and in association with OC use (Figure 3). Plasma AT levels were significantly higher in men than in women without and with OC (*p* = 0.0148 and *p* = 0.001, respectively). α_2_M and fibrinogen were significantly lower in men compared to women without OC (*p* = 0.0146 and *p* = 0.0472, respectively). OC use did not cause any significant difference in plasma AT levels, α_2_M or fibrinogen.

## Discussion

Since the development of the semi-automatic CAT method for the measurement of thrombin generation, computational modeling of thrombin generation, fibrinolysis or more widely the computational modeling of coagulation has been a topic of interest (32–34). Such computational models can vary from complete computational models that generate a thrombin generation curve based on plasma coagulation factor levels and the reaction constants for each reaction in the coagulation cascade (33, 35, 36), to reduced models that only describe a part of the thrombin generation process (37), such as thrombin dynamics analysis. Thrombin dynamics analysis is a hybrid method that uses both the experimentally generated thrombin generation curve, as a computational model to predict the thrombin inactivation rate to compute the prothrombin conversion curve (14). Therefore, TD analysis pinpoints out changes in the hemostatic balance to the pro- or anticoagulant pathway during TG (14, 23). Several studies using TD analysis based on TG data generated by the semi-automated CAT method have shown its clinical relevance (15–22, 30, 38–40).

In healthy individuals, prothrombin conversion parameters PC_tot_ and PC_max_, and anticoagulant parameter TDC, are known to be higher in subjects with a non-O blood group, compared to subjects with blood group O (30). Another study pointed out that strenuous exercise reduces prothrombin conversion in healthy subjects (16), and a study in platelet rich plasma showed that platelets have a significant and positive effect on PC_tot_, PC_max_, T-AT, and T-α2M complex formation (40). Moreover, thrombin dynamics parameters are known to differ in children compared to adults (18). Prothrombin conversion is known to be higher and T-α2M is more in important in children than adults in the balance between T-AT and T-α2M. Furthermore, in the antiphospholipid syndrome, the hemostatic balance is shifted toward a more pro-thrombotic phenotype due to elevated prothrombin conversion but unchanged thrombin inactivation rates (21). Additionally, within the group of APS patients, increased TG and prothrombin conversion were associated with a history of thrombosis (29).

We set out to perform TD analysis using TG curves acquired on the ST Genesia, which is currently available in clinical laboratories. We determined the reference values for all TD parameters using the ST Genesia reagents (STG-BleedScreen, STG-ThromboScreen, and STG-DrugScreen) in a population of 112 healthy donors. The reference ranges should be verified by other laboratories according to the CLSI guidelines in 120 healthy volunteers, if not feasible, it may be acceptable if local measurements on 20 or more healthy subjects yield comparable results (27, 41). Moreover, we investigated the effect of sex and the use of oral contraceptive (OC) on TD parameters.

An additional advantage of TD analysis is that it offers the possibility to perform *in silico* modeling of the changes in TG when plasma coagulation factor levels change. For example, in the past, TD analysis in cirrhosis patients showed that although the PC_tot_ is reduced due to reduced plasma prothrombin levels, PC_max_ is elevated. However, AT levels are lower in cirrhosis patients due to reduced AT production, which results in a rebalanced TG. Furthermore, *in silico* analysis, that was performed as part of thrombin dynamics analysis, pointed out that the choice of prothrombin complex concentrates containing AT are probably safer for treatment of bleeding in liver cirrhosis patients than prothrombin complex concentrates without AT (19). Indeed, this was confirmed by a clinical study performed by the group of Lisman et al. (42), who found that treatment with prothrombin complex concentrates without AT increased TG by 2-4-fold, whereas the administration of fresh frozen plasma, containing both pro- and anticoagulants, increased TG only slightly. Schöchl et al. (43) demonstrated the importance of AT inclusion in prothrombin complex concentrates for treatment in traumatic and surgical coagulopathy. Additionally, the *in silico* prediction of the increase of TG by silencing miRNA targeting AT expression in individual hemophilia patients is expected to improve the dose targeting of the drug (23, 24).

Since the measurement of TG has been fully automatized on the ST Genesia analyzer, TG curves can now also be acquired in clinical laboratories (12, 13, 44–47). Therefore, we investigated TD analysis using ST Genesia TG data to establish normal ranges for TD parameters in an apparently healthy population. The reference values for TD parameters obtained from ST Genesia TG curves are very comparable to the referenced values previously determined for TD parameters obtained from CAT data (23). As mentioned previously, the ST Genesia is based on its precursor the CAT assay. They both use the same thrombin-sensitive substrate, but they differ in reagent content and calibration method. TD parameters determined with the STG-BleedScreen reagent, which contains the lowest amount of TF, correspond well-to TD parameters measured with the PPP Reagent Low used in the CAT method (23). Similarly, TD parameters measured with the STG-ThromboScreen without TM and STG-DrugScreen reagent, containing, respectively an intermediate and a high TF concentration, corresponded well-to TD parameter values obtained with the CAT PPP Reagent and PPP Reagent high, respectively (23).

We also investigated the effect of sex and OC use on TD parameters, as these are known to affect TG (13, 48) and coagulation tests in general (49, 50). In our previous study we already demonstrated differences in women taking OC compared to men and women without OC (13). This is in line with the findings in this study, were we demonstrated that TD parameters did not differ between men and women without OC. The only difference found in TD parameters between men and women is the higher amount of T-a_2_M complexes found in women compared to men. In CAT TD we found similar results, including a (non-significant) trend toward higher T-a_2_M formation in women (23). This discrepancy between men and women can be attributed to the higher level of α_2_M in women compared to men, which stimulates the inhibition of thrombin by α_2_M (23, 51, 52).

On the contrary, the use of OC has distinctive effects on TD parameters, including the attenuation of the inhibitory effect of TM, as known previously from literature (53, 54). The use of OC causes a significant increase in almost all TD parameters, both in ST Genesia- and CAT-based TD analysis, in the absence of TM (23). The use of OC increases TG through the stimulation of both the total amount and the maximal rate of prothrombin conversion (PC_tot_ and PC_max_). This is in line with the fact that OC use has an attenuating effect on the activated protein C pathway, which is a natural anticoagulant system that inhibits the production of thrombin (48–50). Although this effect is more pronounced in the presence of TM, it was also observed in its absence. We have previously shown that plasma prothrombin and FX levels are important influencers of prothrombin conversion, as both PC_tot_ and PC_max_ increase dose-dependently with the prothrombin and FX level (23). Remarkably, the effect of FV levels on PC_tot_ and PC_max_ was marginal around its physiological plasma concentration, indicating that OC use might have additional effects on prothrombin conversion besides the well-known inhibitory effect on the activated protein C pathway (48–50). This could also explain why the effect is also detected in the absence of TM.

In conclusion, we showed that TG curves measured on the fully automated ST Genesia device can be used as an input for TD analysis. The data obtained can be used as reference values for future clinical studies (i.e., after verification of these reference values) as the balance between prothrombin conversion and thrombin inactivation has shown to be useful in several clinical settings. Therefore, the introduction of the ST Genesia to the clinic is a future opportunity to also use the TD method in specified clinical settings.

## Data availability statement

The original contributions presented in the study are included in the article/Supplementary material, further inquiries can be directed to the corresponding author/s.

## Ethics statement

The study was conducted according to the Declaration of Helsinki (2013) and approved by the Medical Ethical Committee of the Maastricht University Medical Centre. Written informed consent was obtained from all participants for their participation in this study.

## Author contributions

AC and BD designed the study. RD collected the samples. MN and QY performed the experiments. MN and RD analyzed the data and wrote the first draft of the manuscript. AC, HT, and BD critically revised the manuscript. All authors contributed to the article and approved the submitted version.

## Conflict of interest

AC is full-time employee of Diagnostica Stago S.A.S. QY, MN, RD, and BD are employees of Synapse Research Institute, part of Diagnostica Stago S.A.S. The remaining author declares that the research was conducted in the absence of any commercial or financial relationships that could be construed as a potential conflict of interest.

## Publisher's note

All claims expressed in this article are solely those of the authors and do not necessarily represent those of their affiliated organizations, or those of the publisher, the editors and the reviewers. Any product that may be evaluated in this article, or claim that may be made by its manufacturer, is not guaranteed or endorsed by the publisher.
