# Supplementary material for: Semi-automated thrombin dynamics applying the ST Genesia thrombin generation assay
Source: Front Cardiovasc Med. 2022 Jul 26;9:912433. doi: 10.3389/fcvm.2022.912433 (PMC9360406; doi:10.3389/fcvm.2022.912433)
Supplement: Supplementary file 1 [file Table_1.docx]

**Supplementary Table 1: Thrombin generation in men and women with and without the use of oral contraceptives.** TG was measured with STG-BleedScreen, STG-DrugScreen and STG-ThromboScreen on the ST Genesia. Data shown are median with IQR (N=112). Abbreviations: IQR, interquartile ranges; OC, oral contraceptives; M, men; W-, women without OC; W+, women with OC; ETP, endogenous thrombin potential; Vel. Index, velocity index; TM, thrombomodulin.
